# A brain-derived metric for preferred kinetic stimuli

**DOI:** 10.1098/rsob.120001

**Published:** 2012-02

**Authors:** Semir Zeki, Jonathan Stutters

**Affiliations:** Wellcome Laboratory of Neurobiology, University College London, London WC1E 6BT, UK

**Keywords:** V5, V3 complex, parietal cortex, medial orbito-frontal cortex, visual motion

## Abstract

We here address the question of whether there is any correlation between subjective preference for simple configurations within a specific visual domain such as motion and strength of activity in visual areas in which that domain is emphasized. We prepared several distinctive patterns of dots in motion with various characteristics and asked humans to rate them according to their preference, before and while scanning the activity in their brains with functional magnetic resonance imaging. For simplicity, we restricted ourselves to motion in the fronto-parallel plane. Moving patterns produced activity in areas V1, V2, the V3 complex (V3, V3A, V3B) and V5, but only in areas V5, V3A/B and parietal cortex did the preferred kinetic patterns produce stronger activity when compared with the non-preferred ones. In addition, preferred patterns produced activity within field A1 of medial orbito-frontal cortex (mOFC), which is not otherwise activated by kinetic stimuli. Hence, for these areas, stronger neural activity correlated with subjective preference. We conclude that configurations of kinetic stimuli that are subjectively preferred correlate with stronger activity within early visual areas and within mOFC. This opens up the possibility of more detailed studies to relate subjective preferences to strength of activity in early visual areas and to relate activity in them to areas whose activity correlates with the subjective experience of beauty.

## 2. Introduction

In the work reported here, we address within a specific and restricted context a more general question of whether there are any definable characteristics of stimuli that render them more attractive, or at any rate preferable. The question has of course been theoretically addressed many times before in artistic speculation, though within a much broader context. Characteristics such as harmony, proportion and symmetry have at various times been considered to be attributes of beautiful works, but without a general consensus. This is perhaps not surprising; attributes such as harmony or proportion are difficult to define for all works that are apprehended as beautiful except in terms of the perceiver. Even the extent to which easily definable properties such as symmetry or proportion, at least for visual objects, are characteristic of beautiful works has been much debated [1]. Within vision, what constitutes proportion or symmetry in one category of visual stimuli (e.g. objects) cannot be easily translated to other attributes (e.g. colour or motion). One way around this difficulty is to concentrate on a single visual attribute, such as visual motion, and enquire whether there are any characteristics or configurations that, for human observers, make some kinetic patterns preferable to others and, if so, whether we can account for this preference in neural terms. Basing ourselves on the functional specialization of the visual brain for different visual attributes [2–4], among which is a specialization for visual motion [5–7], we asked whether there are any particular patterns of dots in motion that stimulate visual areas known to contain directionally selective cells preferentially. Of these, the V5 complex (MT+) is the most prominent, although other areas, such as those comprising the V3 complex (V3, V3A and V3B), which are also dominated by a magnocellular input [3,8], have been shown to contain directionally selective cells [9] or to be responsive to motion in human functional magnetic resonance imaging (fMRI) experiments, though less robustly than V5 [10–17].

## 3. Material and methods

### 3.1. Ethics statement

Informed written consent was obtained from all participants and the University College London (UCL) Research Ethics Committee approved the study.

### 3.2. Subjects

Nineteen healthy subjects (10 males; minimum age 21, maximum age 56, mean age 32) were recruited through advertisements and from the UCL psychology subject pool; three of them were excluded after being scanned because their rating data had not been correctly recorded. None of our participants was an artist and all had normal or corrected-to-normal vision.

### 3.3. Stimuli

Stimuli were generated using Processing (www.processing.org) and then passed to Cogent 2000 and Cogent Graphics (www.vislab.ucl.ac.uk/cogent.php) for playback. Subjects were shown and asked to rate the stimuli twice: once during a visit to the laboratory one or more days before scanning, when the experiment was also explained to them, and once in the scanner.

In the pre-scanning session subjects sat in a darkened room at a fixed distance from a cathode ray tube computer display. They rated the patterns using a computer keyboard. The responses in this part of the study were on an 8-level scale.

Stimuli consisted of eight patterns of moving white dots on a black background, designed with the expectation that some patterns would be preferred to others. The patterns were generated algorithmically using trigonometric functions or particle systems [18], and were matched for the number of dots and their linear velocities at the five velocity levels used. In the pre-scanning experiments, subjects sat at a distance of 60 cm from the display and each dot of the display subtended 0.5**°** of visual angle. In the scanner, subjects were positioned 55 cm from the display, which had a width of 31 cm. The actual area used to display the stimuli was adjusted so that each subject was able to see the entire field of dots. Each individual stimulus contained 192 dots. The speed of the dots’ motion in the pre-scanning sessions was varied in five steps, corresponding to 2.86, 5.73, 8.59, 11.46 and 14.32 deg s^−1^, while speeds in the scanning session varied based on how the stimuli were scaled down for display. The mean dimensions of the area in which the stimuli were shown after individual adjustment were 29 × 23**°**. A single dot subtended a visual angle of approximately 0.17**°,** the size reduction being necessary to make the entire stimulus area visible to the subject in the scanner. The stimuli are available for viewing at www.vislab.ucl.ac.uk/kinetic_beauty_movies. We emphasize that the two patterns preferred by the majority of subjects had different characteristics and both differed in their characteristics from a pattern preferred by one of the other subjects (see below and §5).

Two subjects were given two stimulus sessions in the scanner owing to a recording failure (and only data from the second session was used for them); the remaining subjects were given one session. The session began with a 26 s period with no stimulus on the screen. Brain volumes recorded during this period were discarded from subsequent analysis to allow *T*_1_ equilibration effects to subside. The stimulus sequence began after this period. A block design was used to plan the timing of the stimuli. The patterns were shown in 16 s epochs followed by a 5 s inter-stimulus interval during which the subject was asked via a text prompt to rate the preceding pattern in terms of preference, on a scale of 1 (least preferred) to 4 (most preferred), by pressing a key. Subjects were not given any guidance as to what aspects of the stimuli they should base their ratings on. It was made clear to them that they should rate each stimulus on its own merits rather than relative to the other stimuli and that if they did not have a strong preference for any of the stimuli, they should rate them all neutrally. Once this was done, the screen turned to a mid-grey until the onset of the next epoch. In total, there were 45 epochs, each pattern was shown five times at each of the different speeds (see above). Additionally, there were five epochs during which a static arrangement of dots was shown to provide a baseline of activity for the subsequent analysis. The patterns were ordered using a pseudo-random system which ensured that there were no occasions on which the same pattern was shown in two adjacent epochs.

### 3.4. Scanning details

Scanning was done in a 1.5 T Siemens Magneton Sonata MRI scanner fitted with a head volume coil (Siemens, Erlangen, Germany) to which an angled mirror was attached, allowing subjects to view a screen onto which stimuli were projected using a liquid crystal display projector.

An echo-planar imaging sequence was applied for functional scans, measuring blood oxygen level-dependent (BOLD) signals (echo time TE = 50 ms, repeat time TR = 90 ms, volume time = 4.32 s). Each brain image was acquired in a descending sequence comprising 48 axial slices, each 2 mm thick, with an interstitial gap of 1 mm and a voxel resolution of 3 mm, covering nearly the whole brain. After functional scanning had been completed, a *T*_1_ modified driven equilibrium Fourier transform anatomical scan was performed in the sagittal plane to obtain a high-resolution structural image (176 slices per volume, constant isotropic resolution of 1 mm, TE = 3.56 s, TR = 12.24 s). During scanning, subjects’ eye gaze position, heart rate and respiration were recorded.

### 3.5. Analysis

Data were prepared for analysis in SPM5 [19] using the procedure described by Zeki & Romaya [20]. The onsets and durations of the patterns were modelled as boxcar functions. Head movement parameters calculated from the realignment pre-processing step were included in the model as regressors of no interest. Stimulus functions were convolved with the default SPM5 canonical haemodynamic response function and entered into a linear convolution model (for each subject). Impulse functions convolved with the haemodynamic response were added to the generalized linear model to account for activity related to keypresses. Speed of motion was included as a parametric modulator of no interest in the models. The ratings given by subjects during scanning were included as a modulator. Maximum-likelihood estimates of the associated parameters were then taken to the second (between-subject) level for random effects inference, using the summary statistic approach [21]. This involved taking contrasts or mixtures of parameter estimates summarizing condition-specific effects in each subject and creating statistical parametric maps of unpaired *t*-statistics.

The following contrasts were generated: voxels where viewing a moving pattern produced a greater BOLD response than a stationary one; voxels with a BOLD response proportional to the rating given in the scanner; voxels with a greater BOLD response for patterns rated 4 than for patterns rated 1 in the scanner.

## 4. Results

### 4.1. Determination of the preferred characteristics of the kinetic stimuli from ratings provided

Subjects rated stimuli according to their preferences on a scale of 1–8 during a pre-scanning visit and 1–4 while in the scanner. The rating was given immediately after each stimulus was viewed. Patterns 2 or 5 were the first preference for 13 out of 16 subjects and the second preference for 15 out of 16; hence they were significantly more preferred by subjects (table 1). There were two departures from these preferences, which are significant for the interpretation of results: one subject had a preference for pattern 4, which was not preferred by the others, while another subject did not have a marked preference for any of the patterns. Patterns 2 and 5 differed from one another in smoothness of motion, uniformity of dot distribution and coherence of motion. Pattern 4 also differed from the other patterns, including patterns 2 and 5, on these measures (see electronic supplementary material for a full table of pattern characteristics and an explanation of their calculation).

**Table 1.** The eight patterns and the mean rating (from 1–4; 4 being the most preferred) given to each by subjects when responding in the scanner (*n* = 75).

pattern	mean rating	s.d.
1	1.9	0.75
2	3.4	0.80
3	1.9	0.86
4	2.6	0.67
5	3.2	0.84
6	1.8	0.94
7	2.4	0.81
8	1.9	0.82

### 4.2. Imaging study

In table 2 we report activations that were significant at *p* < 0.05 for family-wise error rate (FWE), with a Bonferroni correction for multiple comparisons and trend-significant activations at *p*_FWE_ < 0.1, corrected (in italics). We also report one small volume correction (SVC) activation based on previous results.

**Table 2.** Activation sites. Activations shown are significant at *p*_FWE_ < 0.05 or *p* < 0.1 (in italics) after Bonferroni correction for multiple comparisons. *k*_E_ is the cluster size in voxels. Coordinates are given in MNI space.

contrast	brain areas	*p*_FWE_	*k*_E_	*X*	*Y*	*Z*
motion > static	calcarine sulcus and surroundings, dorsal and ventral V2 and V3	0	572	21	−96	12
	lV5	0	103	−36	−60	9
	rV5	0	129	48	−57	3
modulated with rating	lV5	0	193	−51	−69	3
	rV3B	0	76	36	−87	−6
	left parietal cortex	0.009	44	−33	−36	45
	right superior parietal lobule	0	130	21	−57	63
	rV5	0.006	48	48	−78	3
	left superior parietal lobule	0	94	−18	−57	63
	*right superior frontal sulcus*	*0.074*	*27*	*21*	−*6*	*54*
liked > disliked (rated 4 > rated 1)	ventral rV3B	0.002	51	36	−84	−6
	lV5	0.001	54	−48	−75	3
	*left occipital/parietal*	*0.096*	*22*	−*21*	−*90*	*30*

#### 4.2.1. Activation of visual motion-related cortex

**Figure 1.**
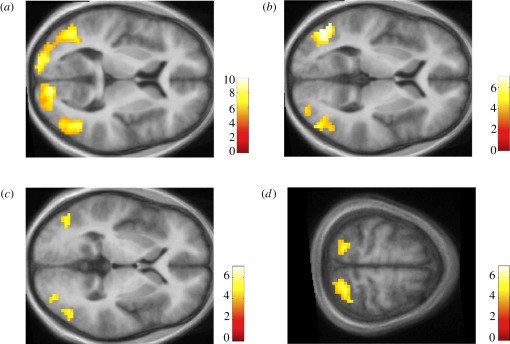
Activation sites. (*a*) Contrast motion > static (background threshold *p*_uncorr_ < 0.0001, cluster threshold *k*_E_ > 0, horizontal section at *z* = 5). (*b*) Visual cortical areas at which activity was parametrically modulated by rating (background threshold *p*_uncorr_ < 0.001, cluster threshold *k*_E_ > 0, horizontal section at *z* = 3). (*c*) Cortical areas from the contrast patterns rated 1 > patterns rated 4 (background threshold *p*_uncorr_ < 0.001, cluster threshold *k*_E_ > 0, horizontal section at *z* = 0). (*d*) Parietal cortex activations that correlated with rating (as in (*b*); background threshold *p*_uncorr_ < 0.001, cluster threshold *k*_E_ > 0, horizontal section at *z* = 63).

As expected, the contrast moving dots > stationary dots led to strong activity in the V5 complex [22,23] and also to a large area of activation in the occipital lobe, which includes the upper and lower lips of the calcarine sulcus (corresponding to area V1), as well as dorsal and ventral V2; the activation also included areas of the V3 complex (V3, V3A and V3B; table 2 and figure 1*a*). All these areas are known to have directionally selective cells [9,24] or to be responsive to motion [25–27]. There was no significant activation of parietal cortex or of medial orbito-frontal cortex (mOFC). There was a trend-significant activation in the right superior frontal sulcus.

#### 4.2.2. Parametrically modulated response

A parametric analysis of the relationship between the BOLD signal and the declared preferences for the moving stimuli showed that cortical activity was positively correlated with subjective preference within the right and left areas V5 and right V3A/B, which are not readily distinguishable from one another even in retinotopic studies (they correspond to LO1 and LO2 in the terminology of Larsson & Heeger [16]; table 2 and figure 1*c*) and bilaterally within the parietal cortex (figure 1*d*), which is also responsive to motion [28,29] and has been implicated in perceptual organization generated by motion or other cues [30,31]. It also led to activation of field A1 of mOFC ((−15, 52, −12) Montreal Neurological Institute (MNI) space with a peak level significance (FWE corrected) of 0.034) when we used an SVC of 18 mm radius, centred on the coordinates ((−3, 41, –8) MNI space) from a previous study of beauty [25]. Speed of motion was not related to the preference expressed. There were no significant activations for second order or higher expansions of the rating.

A parametric analysis for the one subject who preferred pattern 4 to patterns 2 and 5 showed that cortical activity correlated positively with subjective preference within his V5 and within the right V3A/B, but not in the parietal cortex. For the subject who had rated most patterns equally, there were no areas in which strength of activity was proportional to preference, although the pattern of activity in this subject's brain was similar to that of other subjects in the contrast motion > static.

#### 4.2.3. Preferred > non-preferred (4 > 1)

A contrast of preferred more than non-preferred led to the activation of left V5 and right V3A/B, and trend-significant activation in left occipito-parietal cortex (table 2 and figure 1*c*).

We could not detect any significant activations in the contrast non-preferred more than preferred.

## 5. Discussion

Our purpose in this study was to begin an enquiry into the relationship between declared (subjective) preferences for simple visual stimuli on the one hand and brain activation on the other, concentrating specifically on early visual areas. The functional specialization of the visual brain [2–4] allowed us to restrict our enquiry to one domain, that of visual motion. Although any visual or indeed cortical area in which strength of activity correlated with strength of subjective preference would have been of interest, we were especially interested in areas containing directionally selective cells or ones that, in the human, respond strongly to visual motion stimulation. Of these, the most prominent is a set of motion-sensitive visual areas comprising V5 and its satellites (the V5 complex or MT+ [26,27]) and other visual areas such as V3, V3A and V3B, which, though having lower concentrations of directionally selective cells, are nevertheless dominated by a magnocellular input and give robust responses to visual motion stimulation [13,32–34]. As our sole concern was whether it is possible to relate the strength of cortical activity in early visual areas with subjective preference, we did not think it useful, for the present study, to try and subdivide the active areas further with retinotopic mapping or to search for subdivisions or groupings within them for which responses correlate with preference, although both approaches will be of interest for future studies.

### 5.1. Strength of activation and motion-sensitive areas of the cortex

Even though directionally selective cells are most prominently concentrated in area V5 and its satellites (the V5 complex), several other visual areas have been described as containing them. These include areas V1, V2, V3, V3A and V3B, all of which have been responsive to stimulation with visual motion in human fMRI experiments, to varying degrees [12]. Of these, it is area V5 that has been the most extensively studied, with results that are now generally agreed upon. Chief among these are that its cells are overwhelmingly responsive to motion, that the great majority are directionally selective and that most are indifferent to both the colour and the form of the stimulus [5,8,35]. Indeed, many respond optimally to a spot or spots moving in the appropriate direction. It is almost certain that the activity of neurons in V5 is at the basis of the perception of motion in both monkey and human [22,36,37], with the behavioural, psychometric functions and the physiological, neurometric ones from V5 cells in monkeys being almost identical [38]. These characteristics make it relatively easy to prepare visual stimuli that activate V5 strongly. In this study, we opted for patterns of dots in motion in the fronto-parallel plane, known to activate human V5 [30,32], with patterns of incoherently moving dots being more potent activators than a pattern of coherently moving ones [32,39], presumably because more directionally selective cells are activated with incoherently moving patterns.

The areas that were prominently active in the contrast motion more than static were not identical to those in which there was relationship between BOLD signal and preference. In particular, areas V1 and V2 showed no activity related to preference. Activity related to preference was seen only in the V3 complex, V5, parietal cortex and field A1 of mOFC (neither of the latter two areas having shown activity in the contrast motion > static). While the directionally selective cells of V1 respond to component motion of stimuli and those of V5 to their direction of motion [40], the directionally selective cells of areas V2 and V3 have been rather less extensively studied. Area V3 is less directionally selective than V5 in both monkey and human [9,13], but has been found to be responsive to visual motion stimulation in several human studies. Areas V3A and V3B (which are not easily distinguishable from one another even with retinotopic mapping [16,41]) are located dorsally in the brain but represent both upper and lower quadrants [42], also contain directionally selective cells [9] and have been reported, in the human, to be more responsive to motion than V3, though not as responsive as V5 [10,13]. Cells responsive to more complex types of motion, including optic flow patterns, have also been described in both monkey [43,44] and human [28,45] parietal cortex. The latter cortex has in fact been subdivided on the basis of the preferred type of motion [28], but, in the absence of detailed retinotopic mapping, we cannot be certain of which subdivision to allocate our activity to within the parietal cortex. On the basis of MNI coordinates, we would place it as lying closest to IPS3.

That areas outside of V3A and V3B should have lower concentrations of directionally selective cells and be less responsive to simple planar motion than V5 implies, of course, that the preference for particular kinetic configurations may be dictated by a relatively small proportion of cells within it. This would not be surprising. Based on their studies of V5, Shadlen & Newsome [46] estimated that 100 neurons may be the fundamental signalling units of the cortex. This, and the fact that parts of the V5 complex may be more responsive to coherent and others to incoherent motion [23], points to the need for more detailed future studies using techniques such as multi-voxel pattern analysis, which would allow us to determine whether such cells form groupings within areas V5 and V3A/B.

It is difficult to predict from what little is known about the characteristics of motion-selective cells in the activated areas (apart from V5) if there are any particular patterns of moving dots, between coherently moving ones at one end and incoherently moving ones at the other, that are more effective in activating areas containing directionally selective cells than other patterns and, if so, whether they are also the ones that are preferred by human subjects. The notion that such cortical areas might have evolved in response to, and in preference for, particular patterns of motion such as optical flow or biological motion is plausible; this in turn makes it plausible to suppose that the preferred patterns would not only evoke greater activity in V5 and other cortical areas with directionally selective cells, but also lead to the patterns themselves being preferred subjectively.

### 5.2. Preferred kinetic patterns and physical characteristics

In this study, we have shown that, of the visual areas that contain directionally selective cells and respond strongly to visual motion, certain kinetic patterns with definable characteristics, and which are subjectively preferred, evoke more powerful activity only in V5, the areas of the V3 complex and in the parietal cortex, compared with patterns with other definable characteristics or ones lacking them. There are two important issues to address in this context: one is that the recorded subjective preference and the observed accompanying stronger cortical activation in the V3 complex, in V5 and in the parietal cortex are related to preference and not to physical characteristics of the stimulus. The subject who preferred pattern 4 and thus differed from the majority also showed a correlation of cortical activity with subjective preference, even though his preferred kinetic pattern had different physical characteristics from the ones preferred by the majority. As well, another subject who had rated all the stimuli, with their different characteristics, equally had no parametric relation between cortical activity and declared preference. Although all but two of the patterns had some element of grouping of the kinetic dots, which may have suggested objects to some subjects, these groupings differed in size and the extent of the screen occupied, and yet subjects preferred some groupings over others, and one subject preferred pattern 4, which did not exhibit groupings. This makes it very unlikely that grouping dictated preference. All this fortifies the conclusion reached from the other results here, namely that the relationship we observed is indeed between declared subjective preference and cortical activity, rather than with specified characteristics, although of course each subject preferred stimuli with definable characteristics.

### 5.3. The role of attention

It is worth considering next the extent to which our results could be accounted for by attention. Attentional load enhances the strength of activity in V3 and V5 [47,48]. Yet this enhancement is always accompanied by a pattern of cortical activity that includes not only parietal but frontal cortex as well [49–53], and hence a pattern of cortical activity that was not observed in our study. Nor was there activity in V1 or V2, the activity in both of which is modulated by attention [54,55]. Evidence suggests that attentional mechanisms in the parietal cortex are stimulus-driven, whereas the frontal cortex exerts a top-down attentional influence [56,57]. Because the activity we observed is restricted to the parietal cortex, we assume that it is only stimulus-driven. The difficulty in separating attention and preference in a stimulus-driven context, in addition to the fact that all our stimuli had the same number of dots moving at the same speeds and covering the same extent of the field of view, makes it unlikely that the results we observed are owing to attention towards spatial location or to top-down attentional influences from the frontal cortex.

It is to be noted that we did not ask subjects to rate the stimuli according to how beautiful they felt they were; the kind of simple kinetic stimuli that we used are not obviously characterized as beautiful by many, even though kinetic art that remotely echoes our stimuli (e.g. the mobiles of Alexander Calder where movement is emphasized and form and colour are de-emphasized) has given aesthetic satisfaction to writers and poets [58]. For this study, preference seemed to us to be a better and more secure guide of subjective satisfaction, although we are aware that a study based on preference may end up as a prelude to studying those characteristics in visual stimuli that are experienced as beautiful (see below). It is thus encouraging to note that there was activity in field A1 of mOFC [25], even though it could only be elicited with the use of an SVC based on our previous study of this field. Activity in mOFC has been reported to correlate with the experience of beauty, but no study has ever reported it to respond to moving stimuli, leading us to conclude that the activity that we report there is related to preference alone.

It is evident that there is potentially an infinite number of kinetic stimuli that we could have prepared and some could have led to even stronger preferences, perhaps leading even to aesthetic preferences, and stronger activity than what we report. This, however, would have been a very demanding task and would not, in any way, have improved upon the conclusion that we reach here, namely that some kinetic patterns are preferred over others, that there is no constant characteristic of the stimuli that makes them preferable to all subjects, and that preferred kinetic stimuli lead to stronger activity within the motion-sensitive area V5, within the V3 complex of areas and in the parietal cortex. As well, we are aware that variations in the part of the visual field in which the stimuli are presented, as well as variations in the density and duration of dots, could have led to variations in preference, as they do in discrimination thresholds [59]. But to make the study more manageable, we instead opted for a limited number of patterns, and used these to detect preferences and to correlate preferences with the strength of activity in early visual areas.

The results given here edge us closer to understanding the relationship between identifiable characteristics of a kinetic visual stimulus and its aesthetic rating. Whether the approach we have used in this study can, with modifications, be used for other types of visual stimulus, in the domains of form and colour, for example, remains to be seen. For the moment, we restrict ourselves to the conclusion that characteristics of kinetic stimuli can be identified that, when part of the kinetic work, make it preferable over others lacking these characteristics, and that the preference for these stimuli correlates with their capacity to elicit a stronger response from visual areas that are strongly activated by moving visual stimuli as well as from the field A1 of mOFC.

## Supplementary Material

Supplementary data

## References

[1] McManus IC 2005 Symmetry and asymmetry in aesthetics and the arts. Eur. Rev. 13, 157–180 10.1017/S1062798705000736 (doi:10.1017/S1062798705000736)

[2] Zeki SM 1978 Functional specialisation in the visual cortex of the rhesus monkey. Nature 274, 423–428 10.1038/274423a0 (doi:10.1038/274423a0) 97565

[3] Livingstone M Hubel D 1988 Segregation of form, color, movement, and depth: anatomy, physiology, and perception. Science 240, 740–749 10.1126/science.3283936 (doi:10.1126/science.3283936) 3283936

[4] Zeki S Watson JD Lueck CJ Friston KJ Kennard C Frackowiak RS 1991 A direct demonstration of functional specialization in human visual cortex. J. Neurosci. 11, 641–649 2002358 10.1523/JNEUROSCI.11-03-00641.1991 PMC6575357

[5] Zeki SM 1974 Functional organization of a visual area in the posterior bank of the superior temporal sulcus of the rhesus monkey. J. Physiol. 236, 549–573 4207129 10.1113/jphysiol.1974.sp010452 PMC1350849

[6] Zeki S Watson JD Frackowiak RS 1993 Going beyond the information given: the relation of illusory visual motion to brain activity. Proc. R. Soc. Lond. B 252, 215–222 10.1098/rspb.1993.0068 (doi:10.1098/rspb.1993.0068) 8394582

[7] He S Cohen ER Hu X 1998 Close correlation between activity in brain area MT/V5 and the perception of a visual motion aftereffect. Curr. Biol. 8, 1215–1218 10.1016/S0960-9822(07)00512-X (doi:10.1016/S0960-9822(07)00512-X) 9811603

[8] Shipp S Zeki S 2002 The functional organization of area V2, I: specialization across stripes and layers. Vis. Neurosci. 19, 187–210 10.1017/S0952523802191164 (doi:10.1017/S0952523802191164) 12385630

[9] Zeki SM 1978 Uniformity and diversity of structure and function in rhesus monkey prestriate visual cortex. J. Physiol. 277, 273–290 418176 10.1113/jphysiol.1978.sp012272 PMC1282389

[10] Tootell RB Mendola JD Hadjikhani NK Ledden PJ Liu AK Reppas JB Sereno MI Dale AM 1997 Functional analysis of V3a and related areas in human visual cortex. J. Neurosci. 17, 7060–7078 9278542 10.1523/JNEUROSCI.17-18-07060.1997 PMC6573277

[11] Van Oostende S Sunaert S Van Hecke P Marchal G Orban GA 1997 The kinetic occipital (KO) region in man: an fMRI study. Cereb. Cortex 7, 690–701 10.1093/cercor/7.7.690 (doi:10.1093/cercor/7.7.690) 9373023

[12] Smith AT Greenlee MW Singh KD Kraemer FM Hennig J 1998 The processing of first- and second-order motion in human visual cortex assessed by functional magnetic resonance imaging (fMRI). J. Neurosci. 18, 3816–3830 9570811 10.1523/JNEUROSCI.18-10-03816.1998 PMC6793149

[13] Huk AC Ress D Heeger DJ 2001 Neuronal basis of the motion aftereffect reconsidered. Neuron 32, 161–172 10.1016/S0896-6273(01)00452-4 (doi:10.1016/S0896-6273(01)00452-4) 11604147

[14] Zeki S Perry RJ Bartels A 2003 The processing of kinetic contours in the brain. Cereb. Cortex 13, 189–202 10.1093/cercor/13.2.189 (doi:10.1093/cercor/13.2.189) 12507950

[15] Moutoussis K Zeki S 2006 Seeing invisible motion: a human fMRI study. Curr. Biol. 16, 574–579 10.1016/j.cub.2006.01.062 (doi:10.1016/j.cub.2006.01.062) 16546081

[16] Larsson J Heeger DJ 2006 Two retinotopic visual areas in human lateral occipital cortex. J. Neurosci. 26, 13 128–13 142 10.1523/JNEUROSCI.1657-06.2006 (doi:10.1523/JNEUROSCI.1657-06.2006) PMC1904390 17182764

[17] Larsson J Heeger DJ Landy MS 2010 Orientation selectivity of motion-boundary responses in human visual cortex. J. Neurophysiol. 104, 2940–2950 10.1152/jn.00400.2010 (doi:10.1152/jn.00400.2010) 20861432 PMC3007646

[18] Reeves WT 1983 Particle systems: a technique for modelling a class of fuzzy objects. ACM Trans. Graphics 2, 91–108 10.1145/357318.357320 (doi:10.1145/357318.357320)

[19] Friston KA Ashburner JT Kiebel SJ Nichols TE Penny WD 2007 Statistical parametric mapping: the analysis of functional brain images. Amsterdam, The Netherlands: Elsevier

[20] Zeki S Romaya JP 2010 The brain reaction to viewing faces of opposite- and same-sex romantic partners. PLoS ONE 5, e15802 10.1371/journal.pone.0015802 (doi:10.1371/journal.pone.0015802) 21209829 PMC3013131

[21] Holmes WDPA 2007 Random effects analysis. In Statistical parametric mapping: the analysis of functional brain images (eds KJ Friston, JT Ashburner, SJ Kiebel, TE Nichols & WD Penny), pp. 156–165 Amsterdam, The Netherlands: Elsevier

[22] Watson JD Myers R Frackowiak RS Hajnal JV Woods RP Mazziotta JC Shipp S Zeki S 1993 Area V5 of the human brain: evidence from a combined study using positron emission tomography and magnetic resonance imaging. Cereb. Cortex 3, 79–94 10.1093/cercor/3.2.79 (doi:10.1093/cercor/3.2.79) 8490322

[23] Becker HG Erb M Haarmeier T 2008 Differential dependency on motion coherence in subregions of the human MT+ complex. Eur. J. Neurosci. 28, 1674–1685 10.1111/j.1460-9568.2008.06457.x (doi:10.1111/j.1460-9568.2008.06457.x) 18973585

[24] Levitt JB Kiper DC Movshon JA 1994 Receptive fields and functional architecture of macaque V2. J. Neurophysiol. 71, 2517–2542 7931532 10.1152/jn.1994.71.6.2517

[25] Ishizu T Zeki S 2011 Toward a brain-based theory of beauty. PLoS ONE 6, e21852 10.1371/journal.pone.0021852 (doi:10.1371/journal.pone.0021852) 21755004 PMC3130765

[26] Howard RJ Brammer M Wright I Woodruff PW Bullmore ET Zeki S 1996 A direct demonstration of functional specialization within motion-related visual and auditory cortex of the human brain. Curr. Biol. 6, 1015–1019 10.1016/S0960-9822(02)00646-2 (doi:10.1016/S0960-9822(02)00646-2) 8805334

[27] Kolster H Peeters R Orban GA 2010 The retinotopic organization of the human middle temporal area MT/V5 and its cortical neighbors. J. Neurosci. 30, 9801–9820 10.1523/JNEUROSCI.2069-10.2010 (doi:10.1523/JNEUROSCI.2069-10.2010) 20660263 PMC6632824

[28] Orban GA Claeys K Nelissen K Smans R Sunaert S Todd JT Wardak C Durand JB Vanduffel W 2006 Mapping the parietal cortex of human and non-human primates. Neuropsychologia 44, 2647–2667 10.1016/j.neuropsychologia.2005.11.001 (doi:10.1016/j.neuropsychologia.2005.11.001) 16343560

[29] Konen CS Kastner S 2008 Representation of eye movements and stimulus motion in topographically organized areas of human posterior parietal cortex. J. Neurosci. 28, 8361–8375 10.1523/JNEUROSCI.1930-08.2008 (doi:10.1523/JNEUROSCI.1930-08.2008) 18701699 PMC2685070

[30] Braddick OJ O'Brien JM Wattam-Bell J Atkinson J Hartley T Turner R 2001 Brain areas sensitive to coherent visual motion. Perception 30, 61–72 10.1068/p3048 (doi:10.1068/p3048) 11257978

[31] Cusack R Mitchell DJ Duncan J 2010 Discrete object representation, attention switching, and task difficulty in the parietal lobe. J. Cogn. Neurosci. 22, 32–47 10.1162/jocn.2009.21194 (doi:10.1162/jocn.2009.21194) 19199425

[32] Smith AT Wall MB Williams AL Singh KD 2006 Sensitivity to optic flow in human cortical areas MT and Mst. Eur. J. Neurosci. 23, 561–569 10.1111/j.1460-9568.2005.04526.x (doi:10.1111/j.1460-9568.2005.04526.x) 16420463

[33] Moutoussis K Zeki S 2008 Motion processing, directional selectivity, and conscious visual perception in the human brain. Proc. Natl Acad. Sci. USA 105, 16 362–16 367 10.1073/pnas.0802867105 (doi:10.1073/pnas.0802867105) PMC2571003 18843114

[34] Clifford CW Mannion DJ McDonald JS 2009 Radial biases in the processing of motion and motion-defined contours by human visual cortex. J. Neurophysiol. 102, 2974–2981 10.1152/jn.00411.2009 (doi:10.1152/jn.00411.2009) 19759326

[35] Van Essen DC Maunsell JH Bixby JL 1981 The middle temporal visual area in the macaque: myeloarchitecture, connections, functional properties and topographic organization. J. Comp. Neurol. 199, 293–326 10.1002/cne.901990302 (doi:10.1002/cne.901990302) 7263951

[36] Logothetis NK Schall JD 1989 Neuronal correlates of subjective visual perception. Science 245, 761–763 10.1126/science.2772635 (doi:10.1126/science.2772635) 2772635

[37] Rees G Friston K Koch C 2000 A direct quantitative relationship between the functional properties of human and macaque V5. Nat. Neurosci. 3, 716–723 10.1038/76673 (doi:10.1038/76673) 10862705

[38] Britten KH Shadlen MN Newsome WT Movshon JA 1992 The analysis of visual motion: a comparison of neuronal and psychophysical performance. J. Neurosci. 12, 4745–4765 1464765 10.1523/JNEUROSCI.12-12-04745.1992 PMC6575768

[39] Mckeefry DJ Watson JD Frackowiak RS Fong K Zeki S 1997 The activity in human areas V1/V2, V3, and V5 during the perception of coherent and incoherent motion. Neuroimage 5, 1–12 10.1006/nimg.1996.0246 (doi:10.1006/nimg.1996.0246) 9038280

[40] Movshon JA Newsome WT 1996 Visual response properties of striate cortical neurons projecting to area MT in macaque monkeys. J. Neurosci. 16, 7733–7741 8922429 10.1523/JNEUROSCI.16-23-07733.1996 PMC6579106

[41] Press WA Brewer AA Dougherty RF Wade AR Wandell BA 2001 Visual areas and spatial summation in human visual cortex. Vision Res. 41, 1321–1332 10.1016/S0042-6989(01)00074-8 (doi:10.1016/S0042-6989(01)00074-8) 11322977

[42] Essen DC Zeki SM 1978 The topographic organization of rhesus monkey prestriate cortex. J. Physiol. 277, 193–226 418173 10.1113/jphysiol.1978.sp012269 PMC1282386

[43] Schaafsma SJ Duysens J Gielen CC 1997 Responses in ventral intraparietal area of awake macaque monkey to optic flow patterns corresponding to rotation of planes in depth can be explained by translation and expansion effects. Vis. Neurosci. 14, 633–646 10.1017/S0952523800012608 (doi:10.1017/S0952523800012608) 9278993

[44] Siegel RM Read HL 1997 Analysis of optic flow in the monkey parietal area 7a. Cereb. Cortex 7, 327–346 10.1093/cercor/7.4.327 (doi:10.1093/cercor/7.4.327) 9177764

[45] Beer AL Watanabe T Ni R Sasaki Y Andersen GJ 2009 3D surface perception from motion involves a temporal-parietal network. Eur. J. Neurosci. 30, 703–713 10.1111/j.1460-9568.2009.06857.x (doi:10.1111/j.1460-9568.2009.06857.x) 19674088 PMC2902171

[46] Shadlen MN Newsome WT 1994 Noise, neural codes and cortical organization. Curr. Opin. Neurobiol. 4, 569–579 10.1016/0959-4388(94)90059-0 (doi:10.1016/0959-4388(94)90059-0) 7812147

[47] O'Craven KM Rosen BR Kwong KK Treisman A Savoy RL 1997 Voluntary attention modulates fMRI activity in human MT-Mst. Neuron 18, 591–598 10.1016/S0896-6273(00)80300-1 (doi:10.1016/S0896-6273(00)80300-1) 9136768

[48] Chawla D Rees G Friston KJ 1999 The physiological basis of attentional modulation in extrastriate visual areas. Nat. Neurosci. 2, 671–676 10.1038/10230 (doi:10.1038/10230) 10404202

[49] Buchel C Josephs O Rees G Turner R Frith CD Friston KJ 1998 The functional anatomy of attention to visual motion. A functional MRI study. Brain 121, 1281–1294 10.1093/brain/121.7.1281 (doi:10.1093/brain/121.7.1281) 9679780

[50] Kastner S Pinsk MA De Weerd P Desimone R Ungerleider LG 1999 Increased activity in human visual cortex during directed attention in the absence of visual stimulation. Neuron 22, 751–761 10.1016/S0896-6273(00)80734-5 (doi:10.1016/S0896-6273(00)80734-5) 10230795

[51] Kastner S Ungerleider LG 2000 Mechanisms of visual attention in the human cortex. Annu. Rev. Neurosci. 23, 315–341 10.1146/annurev.neuro.23.1.315 (doi:10.1146/annurev.neuro.23.1.315) 10845067

[52] Saygin AP Sereno MI 2008 Retinotopy and attention in human occipital, temporal, parietal, and frontal cortex. Cereb. Cortex 18, 2158–2168 10.1093/cercor/bhm242 (doi:10.1093/cercor/bhm242) 18234687

[53] Zanto TP Rubens MT Thangavel A Gazzaley A 2011 Causal role of the prefrontal cortex in top-down modulation of visual processing and working memory. Nat. Neurosci. 14, 656–661 10.1038/nn.2773 (doi:10.1038/nn.2773) 21441920 PMC3083493

[54] Munneke J Heslenfeld DJ Theeuwes J 2008 Directing attention to a location in space results in retinotopic activation in primary visual cortex. Brain Res. 1222, 184–191 10.1016/j.brainres.2008.05.039 (doi:10.1016/j.brainres.2008.05.039) 18589405

[55] Watanabe M Cheng K Murayama Y Ueno K Asamizuya T Tanaka K Logothetis N 2011 Attention but not awareness modulates the bold signal in the human V1 during binocular suppression. Science 334, 829–831 10.1126/science.1203161 (doi:10.1126/science.1203161) 22076381

[56] Ruff CC Bestmann S Blankenburg F Bjoertomt O Josephs O Weiskopf N Deichmann R Driver J 2008 Distinct causal influences of parietal versus frontal areas on human visual cortex: evidence from concurrent TMS-fMRI. Cereb. Cortex 18, 817–827 10.1093/cercor/bhm128 (doi:10.1093/cercor/bhm128) 17652468 PMC2601025

[57] Ruff CC Blankenburg F Bjoertomt O Bestmann S Weiskopf N Driver J 2009 Hemispheric differences in frontal and parietal influences on human occipital cortex: direct confirmation with concurrent TMS-fMRI. J. Cogn. Neurosci. 21, 1146–1161 10.1162/jocn.2009.21097 (doi:10.1162/jocn.2009.21097) 18752395 PMC2667814

[58] Zeki S Lamb M 1994 The neurology of kinetic art. Brain 117, 607–636 10.1093/brain/117.3.607 (doi:10.1093/brain/117.3.607) 8032869

[59] Moyshon DA 1989 Spatial and temporal summation in the detection of motion in stochastic random dot displays. Invest. Ophthalmol. Vis. Sci. 30, 72–88

