# Supplementary material for: A brain-derived metric for preferred kinetic stimuli
Source: Open Biol. 2012 Feb;2(2):120001. doi: 10.1098/rsob.120001 (PMC3352092; doi:10.1098/rsob.120001)
Supplement: Supplementary data [file rsob120001-s1.doc]

# Supplementary data

## Pattern characteristics

| **Pattern** | **Disuniformity** | **Incoherence** | **Roughness** |
| --- | --- | --- | --- |
| 1 | 1.62 | 0.03 | 2.35 |
| 2 | 9.3 | 2.25 | 1.44 |
| 3 | 2.51 | 0.03 | 3.26 |
| 4 | 2.28 | 3.28 | 2.91 |
| 5 | 3.48 | 1.63 | 0.18 |
| 6 | 2.13 | 0.07 | 3.25 |
| 7 | 2.3 | 0.14 | 3.39 |
| 8 | 2.42 | 0.05 | 3.26 |

### Calculation of pattern characteristics

#### Disuniformity

The uniformity was calculated by dividing each frame of the pattern into a grid of 120x80 pixels and calculating the number of dots within each grid cell. The variance of the dot counts in each frame was then calculated and the mean of these variances taken to give the uniformity. Higher disuniformity indicates that the dots were more unevenly distributed.

#### Incoherency

The incoherence was derived by converting the motion between frames of each dot into a direction of motion and then calculating the variance across dots for each frame transition. The mean variance for all frame transitions was calculated to give the final value.

#### Roughness

The roughness was derived by converting the motion between frames of each dot into a direction of motion and then calculating the variance across frames for each dot. The mean variance for all dots was then calculated to give the final value.
